# Integrating basic science in academic cardiology training: two international perspectives on a common challenge

**DOI:** 10.1007/s00392-018-1294-3

**Published:** 2018-06-09

**Authors:** Michael F. Bode, Ingo Hilgendorf

**Affiliations:** 1 Division of Cardiology, Department of Medicine, University of North Carolina at Chapel Hill, Chapel Hill, NC USA; 2 Department of Cardiology and Angiology I, University Heart Center Freiburg-Bad Krozingen, Bad Krozingen, Germany; 3 Faculty of Medicine, University of Freiburg, Freiburg, Germany

**Keywords:** Clinician scientist, Education, Training

## Abstract

Political bodies and professional societies acknowledge that translational research benefits from researchers trained in both, clinical medicine and basic science. Yet, few physicians undergoing clinical training in cardiology seek this dual career (Milewicz et al. J Clin Invest 125:3742–3747, 2015). The reasons are likely manifold, but with cardiology having become increasingly interventional and facing economic pressure, how much attention, credit, and encouragement is given to physicians interested in basic cardiovascular science? Having studied and worked in hospitals and laboratories, in both Germany and the USA, we aim to compare in this article how basic science education is currently integrated into cardiology training at German and US university hospitals, from medical school to more advanced career stages. By doing so, we hope to provide some outside perspectives to young physicians and decision makers alike, that may inspire changes to curricula in the respective countries and around the world.

## Getting an appetite

During undergraduate studies and the first years of medical school, future physicians are first exposed to basic life sciences in theory with limited practice. To earn a doctoral degree in medicine, German medical students need to conduct research and write a dissertation thesis. Two out of three students decide to do so [2]. Some engage in clinical research, and about 40–60% choose a basic scientific research topic [3]. The doctoral supervisor and student agree on the time effort the student is willing to commit to research. This may mean anything from working in the lab while continuing one’s medical studies to interrupting medical studies for full-time research for a year. Some students even choose to go abroad. The study curriculum at German medical schools allows for this kind of flexibility. In fact, the German Cardiac Society, the Society of Internal Medicine and several foundations offer stipends to medical students for up to 1 year to support their scientific endeavors. The MD thesis is a valuable opportunity for German medical students to gain first-hand experience in basic scientific work short of attending a full MD/PhD program offered by some medical and biology faculties and requiring considerable additional time commitment of typically 3–4 years. Students exposed to basic science during their medical studies are, based on our experience and a recent survey, more likely to pursue a medical career at an academic center that offers research opportunities. Overall, 20% of MD students in Germany express an interest in continuing scientific work during their postgraduate training, but less than 5% in basic research [2, 3].

In the US, the first opportunity for students to engage in basic science often presents itself during undergraduate studies or after graduation from college. At this point, it is not difficult to get accepted at a basic research lab to participate in working on a project, and no extensive prior knowledge is required. By engaging in research, students are also able to increase their chances of acceptance to the college or medical school of their choice. During medical school, the study curriculum typically does not allow for dedicated research time for basic science. Therefore, MD/PhD programs to train future physician–scientists have been established at over 90 universities [4]. These programs take an additional 3–4 years compared to a regular MD program, but stipends are often offered. This is an important distinction from the German medical education system, where generally no student fees apply, and student loans and financial burden are therefore less of a concern. After graduation from medical school, students typically enter residency training immediately. Only 6% of medical students have enrolled in MD/PhD programs in recent years, but 83% of those chose academic careers as compared to 16% of other medical school graduates. They were also 3–4 times more successful in receiving NIH grants [5, 6].

## Protected time during training

Clinical training in internal medicine and cardiology at German university hospitals encompasses a common trunk of 3 years of practice in general internal medicine and 3 years in cardiology to qualify for board certification. Obtaining the postdoctoral lecturing qualification (habilitation) for an academic position requires proof of teaching and scientific publication output. The clinical curriculum for board certification, however, does not allocate time for research. Traditionally, department heads had organized protected time and budgets for research for their interested fellows through cross funding. Two-thirds of cardiovascular research in Germany still rely on institutional funding [7]. However, clinical cardiology departments at university hospitals, especially those that underwent privatization, are facing increased economic pressure and constraints that limit their financial leeway. Public funds and medical reimbursement hardly compensate higher demands at academic centers arising from teaching obligations and clinical cases with higher morbidity. Therefore, interested fellows may alternatively apply for part- or full-time funding of their position at their medical faculty, if such programs exist, or for external funding, e.g., by the German Research Foundation, German Center for Cardiovascular Research, German Cardiac Society and Society of Internal Medicine to secure dedicated time for research. Proof of substantial postdoctoral research experience is usually required to qualify for such programs that support postdocs at their home institutes. To gain such experience young physicians apply at the very same organizations and other foundations for a 2–3-year postdoctoral fellowship at a different host institute in or outside Germany while interrupting their clinical training. In life sciences, 60% of stipend holders from the German Research Foundation join a laboratory in the US. Fully committed to science and aware of the time constraints this is typically an intense and highly productive time when young German doctors build scientific expertise in their field of interest and forge long-lasting alliances and networks. For those who live and work abroad this time is also personally stimulating and a defining cultural experience.

As mandated by the American Board of Internal Medicine (ABIM), clinical training to become a cardiologist in the US generally consists of 3 years of internal medicine residency and an additional 3 years of cardiovascular disease fellowship after which the physician qualifies for cardiology board certification. Residency and fellowship curricula are controlled by the Accreditation Council for Graduate Medical Education (ACGME). Although ACGME has recently been trying to incorporate more flexibility into training programs to allow for more research time during training, very few programs actually offer sufficient research time to conduct basic research. Most research opportunities are tailored towards clinical research that can be completed within a few weeks or months. The ABIM therefore offers two alternative pathways. One approach is completion of the regular 3-year residency and application to a 4-year cardiology fellowship that consists of 2 years of protected research time and 2 years of clinical cardiovascular disease fellowship training. A more integrated approach is offered by several of the primarily research-oriented institutions: the ABIM Research Pathway allows for an abbreviated residency training of 2 years, an abbreviated cardiovascular disease fellowship of 2 years, and 3 years of protected research time. While this appears to be the ideal pathway for the dedicated physician–scientist, the commitment to join the program has to be made early on, either prior to residency application or during the first year of residency, and the whole 7-year pathway has to be completed without interruption to be certified in internal medicine and cardiology. Benefits of this pathway oftentimes include funding through NIH T32 grants and various forms of additional support by the institution. In addition, trainees will be able to apply for grants from AHA, NIH, or other sources such as private foundations. Towards the end of the fellowship, trainees can apply for transition grants, such as the NIH K grants, that provide funding for scientists as they become independent researchers under the supervision of a mentor. K08 grants support basic and translational science, while K23 grants sponsor clinical science. The American Physician–Scientists Association lists 31 US institutions that offer a Research Residency and Fellowship Program [8].

## Career options

After finishing a postdoctoral fellowship in basic science, most German physicians will go on to complete their clinical training in cardiology. As mentioned above, they may apply for funding from several sources to establish their own research group. Although still limited in numbers, physician–scientist programs have been established at many German universities, and financial support for pursuing both a scientific and clinical career in parallel is offered by different professional societies. The German Research Foundation and the European Research Council offer Elite Programs for those who wish to primarily dedicate themselves to science. The German Center for Cardiovascular Research, representing 28 sites throughout Germany, launched the Young DZHK program to support students, scientists and physicians alike, through postdoc start-up grants, clinician scientist programs, junior research groups, and even 17 professorships. Yet, young physicians will embrace these possibilities not only for the love of science, but also because they long for new, financially attractive career options. Apart from the traditional positions in institutes of pharmacology and physiology, a growing number of professorships and institutes of molecular or experimental cardiology, that affiliate with clinical departments, arise at academic centers. 16 of the 36 university hospitals currently feature one or more of these positions. Offering opportunities to practice part time in clinical cardiology and basic cardiovascular science, these relatively new positions on the German research landscape have yet to prove whether they are the right tool to ultimately attract more talented cardiologists to basic science. However, engaging in research even advanced careers of those who continue to work primarily in clinical routine as attending physicians and chiefs at academic cardiology departments in Germany, Professor Ali El-Armouche, head of the newly founded “Clinician Scientist Task Force” of the German Cardiac Society, cites a recent survey he led and is currently analyzing.

In the US, cardiologists who wish to remain active in basic cardiovascular research most importantly have to decide how much of their effort they would like to dedicate to basic research or clinical duties. A majority of 70% of physician–scientist members of the American College of Cardiology (ACC) in their early careers devote less then 40% of their time to research. Overall, a quarter of early career academic cardiologists engage in basic and translational science [9]. Clinically, practicing general or interventional cardiology at an academic center are both possible. A higher percentage of effort for basic research means that physicians are more dependent on grants for salary support and have less clinical time, but on the other hand are eligible for more grants from NIH and AHA, and may be more competitive when applying for the same grants as scientists. However, the success rates of ACC members with substantial research commitment (40–75% of time) with regard to securing NIH grants declined by about 50% between 2013 and 2016 despite overall increased NHLBI funding [9]. Lack of time, lack of proper mentoring, and lower incomes than those of full-time clinicians are often criticized in this context. A majority of US cardiologists claimed to lose more than $75,000/year in earnings when choosing an academic career with research, as compared to German and other international ACC members that reported no or minor financial disadvantages [9]. Finding an institution that is supportive of one’s research is key. Despite these apparent challenges, physician–scientist cardiologists are highly searched for at many large academic medical centers and have excellent career options based on their ability to draw ideas from patient care and integrate basic and clinical knowledge to create new approaches to treating disease.

## Conclusions

Physician–scientists in the US and Germany experience many similar and also some particular rewards and obstacles during their careers [7, 10] (Table 1). Learning to balance time commitments between clinical and research duties is essential while competing with full-time researchers on the one hand, and full-time clinicians on the other, for grants and positions. Both in Germany and in the US, a high level of personal commitment is required of the physician–scientist as many universities offer insufficient incentives and support particularly for basic research. When research is supported, it is oftentimes clinical research that promises quicker rewards, practical combination with clinical duties, and cooperation with the industry. Clinical and administrative responsibilities, increasing the referral base, increasing patient volume, decreasing cost per case, and streamlining inpatient services are more likely to be incentivized by department leaders than basic research and teaching that lead to less tangible immediate financial rewards. However, basic cardiovascular research at universities is needed to ensure continued substantial progress in understanding disease processes and developing cures. President-elect of the German Cardiac Society, Professor Andreas Zeiher, in an interview at the 2018 annual meeting of the society therefore declared to advocate for more research and basic science in particular, as an integral part of academic cardiology training.

**Table 1:** Benefits and challenges of German and US curricula for physician–scientists in cardiology

Career steps	Germany	USA
Getting an appetite	+ Medical students can write a MD thesis in less time than a full PhD − Highly variable scientific quality of MD thesis	+ Many students earn a bachelor degree in life sciences prior to attending Medical School − Basic research during medical school is typically restricted to MD/PhD programs
Protected time during training	Postdoc fellowships and physician–scientist programs offered by universities and foundations + Flexible in terms of timing of application and combination with clinical training + International experience − Lower income than clinicians	American Board of Internal Medicine certified combined clinical/science education tracks + Structured training; fellowship spot and some funding often guaranteed − Less flexible, early dedication to such a program required
Career options	+ Growing number of professorships for physician–scientists, some with dual appointment in clinics − Challenge to balance research and clinical duties, especially when being interventional	+ More grants for salary support + More flexible solutions in regards to clinical obligations − Salary more susceptible to cuts in public research funding − Large income gap to full-time clinicians

In Germany, higher flexibility of the medical school and clinical curricula, and multiple easily accessible funding opportunities support early careers in basic science more effectively than in the United States. Furthermore, international exchange is more often sought for. At more advanced career stages, however, German cardiologists face particular challenges. Since in- and out-patient care are divided between hospitalists and general cardiologists in private practice, those that wish to remain in academia and science are often expected to become interventionalists, requiring additional commitment to clinical training. At this level, compared to the USA, there are less opportunities to secure funding for one’s own position and protected time for research. Slowly, some German universities are trying to overcome these shortcomings by offering long-term dual career positions to physician–scientists, but often, these efforts are counteracted by the growing economic pressure and the emphasis on clinical and interventional skills. In the USA, the incorporation of more flexible curricula into the traditional educational system may help to attract more MD students and young residents to basic cardiovascular research, thereby increasing the group of candidates for becoming a physician–scientist in the field of cardiology and minimizing attrition rates. Otherwise, high tuitions for lengthy MD/PhD programs remain an obstacle. In addition, at later career stages, financial disadvantages may discourage cardiologists to enter an academic, research intensive career path.

“Faculty, academic medical center leaders, and professional organizations must continue to strive to foster an environment that furnishes a viable and attractive career path for physician–scientists engaged in basic research. By the same token, physician–investigators must strive to reach beyond the ‘Ivory Tower’ of the research laboratory and work with more clinically oriented colleagues to help close the gap in translation. We need to engage to fulfill society’s expectation that investment in basic research will yield palpable progress in human health beyond increasing fundamental knowledge”, states Peter Libby, Mallinckrodt Professor of Medicine at Harvard Medical School and Brigham and Women’s Hospital cardiovascular specialist. After all, it is the very combination of a scientific mind with clinical skills that embodies academic cardiology. When valued by academic centers and societies, students and residents are more likely to embark on this pathway. The reward is a stimulating career at the crossroads of biologic discoveries and clinical innovation that determine the future of cardiology.
